# Analysis of Ani s 7 and Ani s 1 allergens as biomarkers of sensitization and allergy severity in human anisakiasis

**DOI:** 10.1038/s41598-020-67786-w

**Published:** 2020-07-09

**Authors:** Leticia de las Vecillas, Pedro Muñoz-Cacho, Marcos López-Hoyos, Vittoria Monttecchiani, Victoria Martínez-Sernández, Florencio M. Ubeira, Fernando Rodríguez-Fernández

**Affiliations:** 1 Department of Allergy, Marqués de Valdecilla University Hospital-Instituto de Investigación Marqués de Valdecilla (IDIVAL), Santander, Spain; 2 Department of Immunology, Marqués de Valdecilla University Hospital-Instituto de Investigación Marqués de Valdecilla (IDIVAL), Santander, Spain; 3 Gerencia Atención Primaria, Servicio Cántabro de Salud, Santander, Spain; 4 Department of Microbiology and Parasitology, Faculty of Pharmacy, University of Santiago de Compostela, Santiago de Compostela, Spain; 5 Instituto de Investigación en Análisis Químicos y Biológicos (IAQBUS), Universidad de Santiago de Compostela, Santiago de Compostela, Spain

**Keywords:** Immunology, Microbiology, Biomarkers, Molecular medicine

## Abstract

The high frequency of infection by *Anisakis simplex* (*A. simplex*) has led to an increase in IgE sensitization, turning allergy to this parasite a relevant contemporary health problem. Improving the lack of conventional diagnosis test specificity is crucial to better understand these clinical scenarios. Specific IgE (sIgE) to *A. simplex* extract by ImmunoCAP (*Anisakis*-sIgE) was determined in sera from 403 blood donors (BD) from Cantabria (North of Spain) of which 51 subjects resulted sensitized. Among these latter, 47 were asymptomatic (sABD). The values of total IgE, prick-test, *Anisakis*-sIgE, and sIgE to Ani s 1 (anti-rAni s 1) and Ani s 7 (anti-rAni s 7) were compared between 46 sABD and 49 *A. simplex* allergic patients. The IgE seroprevalence by ImmunoCAP among BD was 12.65%. Allergic patients and sABD showed significant differences in all serum biomarkers evaluated. The area under the curve was assessed for *Anisakis*-sIgE (0.892), sIgE-rAni s 1 (0.672) and sIgE-rAni s 7 (0.668). After a severe reaction, significantly higher levels of *Anisakis*-sIgE and sIgE anti-rAni s 1 were detected. Determinations of sIgE by ImmunoCAP, Ani s 1 and Ani s 7 presented different sensitization patterns between allergic and asymptomatic individuals. The Ani s 1 allergen arises as a possible biomarker to detect patients at risk of suffering severe allergic reactions.

## Introduction

*Anisakis simplex* (*A. simplex*) is a nematode belonging to the Anisakidae family in whose life cycle participate fish, crustaceans and marine mammals^1^. Humans can become an incidental host due to the ingestion of raw or uncooked fish harboring third-stage larvae^2^. In the last decades the prevalence of infected fish by *A. simplex* has increased dramatically and in parallel, health problems associated with this parasite becoming a public health concern^3–6^. In the center and the northern areas of Spain, there is a tendency of consuming fresh fish either raw or prepared using techniques requiring light cooking (e.g., hake and anchovies) which favors the contact with the live larvae^7–9^.

It is generally accepted that an infection by *Anisakis* spp. larvae is required to produce symptoms in humans^10,11^. After a first penetration of the gastrointestinal mucosa by live *Anisakis* larvae, the released antigens induce the production of IgE antibodies in response to the parasite infection^7,12–14^. In subsequences exposures to the larvae, sensitized individuals can develop allergic IgE-mediated symptoms some minutes to hours after the intake of parasitized fish^15^. Clinical manifestations can range from mild to moderate, (such as urticaria, angioedema, bronchospasm) or even anaphylaxis^13^. The local mucosa damage produced by alive larvae may also induce gastrointestinal symptoms, such as dyspepsia, vomiting, abdominal pain^12,15,16^. When these type of clinical features are associated to IgE-mediated symptoms, patients suffer a gastro-allergic anisakiasis^7,17,18^. *Anisakis simplex* sensitization has been also considered as a triggering and/or worsening factor of other diseases such as urticaria/angioedema and dyspepsia^13,19,20^.

Due to the low specificity of traditional test such as skin prick test (SPT) and *Anisakis*-sIgE (ImmunoCAP)^21^, several authors proposed the use of component-resolved diagnosis (CRD) to investigate which allergens are responsible for a given allergic reaction. CRD is a relevant diagnostic tool that can provide relevant information on severity risks and can guide allergists on the management of allergic patients^17,22–25^.

Fourteen *A. simplex* allergens have been described and classified in three groups according to its origin: excretory/secretory, somatic and cuticular proteins^7,17,26–28^. The first group includes relevant major allergens as Ani s 1 and Ani s 7, which are frequently used to measure sIgE responses to *A. simplex* in different populations including non-symptomatic blood donors, fish processing workers and patients suffering gastroallergic anisakiasis or chronic urticaria^9,17,29^.

Recently, Viñas et al.^30^ reported that the presence of IgG and IgE antibodies to Ani s 1 and Ani s 3 allergens in serum can aid to differentiate between patients with and without urticaria in regions where *Anisakis* infections are frequent. Also, cross reactivity between *A. simplex* allergens and those from house dust mites (HDM) or shellfish among others has been proposed as the origin of positive sIgE to complete *A. simplex* extract (ImmunoCAP) in asymptomatic population^31,32^.

However, the role of Ani s 1 and Ani s 7 allergens as biomarkers of severity in *Anisakis*-induced acute allergic reactions were never evaluated. To address this issue, we compared the anti-Ani s 1 and anti-Ani s 7 sIgE responses in a population of sABD with that of a population of patients presenting acute allergic symptoms to this nematode.

## Results

### Population characteristics: demographics and consumption habits

Among 403 BD randomly selected, 381 referred to be asymptomatic when consuming fish and 47 of them presented with *Anisakis*-sIgE > 0.35 KUA/L (sABD) (See Supplementary Fig. S1 online). Gender and age were the only demographic variables with significant differences between allergic group and sABD (*p* < 0.001 and *p* = 0.01, respectively) (Table 1). Based on the anonymous questionnaire answers, the overall frequency of fish consumption did not show significant differences between groups (*p* 0.142). As expected, raw fish intake (57.4% in allergic vs. 31.1% in sABD; *p* = 0.011) and restaurant fish consumption (28.3% in allergic vs. 2.8% in sABD; *p* = 0.002) was more frequent in allergic patients (see Supplementary Table S1 online).

**Table 1:** Demographics and fish consumption habits. Allergic patients and sensitized asymptomatic blood donors to *Anisakis simplex* (sABD).

	Allergic patients (n = 49)	sABD (n = 46)	*p* value
**Demographics**			
Females, %	60.4	25	< 0.001*
Age^a^, year^b^	54.96 (11.63)	48.32 (12.78)	0.010*
Coastal residency^a^	83.7%	81%	0.734
Occupation related to fishery	12.9%	5.7%	0.311
**Fish consumption habits**			
Frequency, n (%)	No significant differences		
Conservation, n (%)	No significant differences		
Restaurant consumption, n (%)	13 (28.3%)	1 (2.8%)	0.002*
Raw preparation, n (%)	8 (16.7%)	1 (2.3%)	0.020*
Species, n (%)	No significant differences		
Fish farm source, n (%)	7 (17.5%)	11 (44.0%)	0.021*
Tolerance of frozen fish	42 (91.3%)	44 (100%)	0.045*
Raw fish consumption	27 (57.4%)	14 (31.1%)	0.011*
Homemade fish-preserves	No significant differences		

Data extracted from an anonymous questionnaire. ^a^Information related to age and residency was collected in all allergic patients and in 44 and 42 of sABD, respectively. Occupation was registered in 31 allergic patients and in 35 ABD. ^b^Mean (standard deviation). *Significant differences.

Thirty-one sABD accepted a follow up consultation 2 years after their recruitment. Most of them were still asymptomatic when eating fish except one of them who experienced an anaphylactic reaction after eating infected fish (undercooked see bass), 1 year after the inclusion date (see Supplementary Fig. S1 online).

### Prevalence of *Anisakis simplex* sensitization and conventional diagnosis test results

Considering the population of 403 BD, the sIgE seroprevalence measured by ImmunoCAP (sIgE > 0.35 kUA/L) was 12.65% (*n* = 51). Median values of total IgE and *Anisakis*-sIgE in the allergic group were 366 IU/mL and 37.80 kUA/L, respectively; and 35.15 IU/mL and 1.79 kUA/L in the sABD. These differences were statistically significant between both groups (*p* < 0.001) (Table 2a). *Anisakis*-sIgE distributed by classes revealed that the majority of the allergic patients (91.9%) presented with levels greater than 3.5 kUA/L (Class 3–6), the 79.6% above 8 kUA/L, the 73.5% above 10 kUA/L and the 63.2% above 17.5 kUA/L (see Supplementary Fig. S2a online). In the sABD group all IgE values were ≤ Class 4 and the 91.3% of which were below 17.5 kUA/L (Class 1–3) the 83.4% below 8 kUA/L and 67.4% below 3.5 kUA/L (see Supplementary Fig. S2a online).

**Table 2:** Results of conventional and component-resolved diagnosis test. (a) Comparison of median values (IQR) between allergic patients and sABD. (b) Comparison of positives results in component-resolved diagnosis test. (c) Sensitivity and specificity of Trisakis 170 (rAni s 1, rAni s 7, or both) considering only allergic patients as true positives.

	Allergic patients (n = 49)	sABD (n = 46)	*p*
**(a)**			
Total IgE (IU/mL)	366 (105–692)	35.15 (14.67–116.25)	< 0.001*
*Anisakis*-sIgE (kUA/L)	37.80 (9.01–88.75)	1.79 (0.79–5.50)	< 0.001*
rAni s 1 (OD)	1.47 (0.22–1.69)	0.13 (0.01–1.30)	< 0.005*
rAni s 7 (OD)	1.34 (0.77–1.67)	0.57 (0.18–1.40)	< 0.005*
SPT (mm)	5.50 (4.50–9.00)	5.00^a^ (2.87–6.12)	0.074
**(b)**			
Positive rAni s 1, n (%)	38 (77.5%)	24 (52.2%)	0.009*
Positive rAni s 7, n (%)	47 (95.9%)	41 (89.1%)	0.206
Double positive	38 (77.6%)	24 (52.2%)	0.009*

	Sensitivity	Specificity	PPV	NPV
**(c)**				
rAni s 1	61.3%	66.7%	77.5%	47.8%
rAni s 7	95.9%	10.9%	53.4%	71.4%
Double positive	47.8%	77.5%	61.3%	66.7%

Cut-off values: *Anisakis*-sIgE > 0.35 kU_A_/L; SPT ≥ 3 mm; rAni s 1 = 0.09 (OD); rAni s 7 = 0.05 (OD). ^a^SPT were performed in 21 out of 46 sABD. OD, optical density. *Significant differences.

All allergic patients had a positive SPT (5.50 mm) and 13 out of 21 sABD tested (76.2%) were also positive (median 5.00 mm) with no significant differences between groups (*p* 0.074) (Table 2a).

### Component-resolved diagnosis test results

The sera from all allergic patients (*n* = 49) and 46 sABD were analyzed using the Trisakis-170 ELISA kit to detect specific anti-rAni s 1 and anti-rAni s 7 sIgE antibodies. Considering the group of allergic patients, 38/49 (77.5%) and 47/49 (95.9%) sera tested positive for rAni s 1 and rAni s 7, respectively. Two sera negative for rAni s 7 tested also negative for rAni s 1 (double negatives). Three sera (6.1%) from the rAni s 7 positive allergic population (ODs = 0.12, 0.31 and 0.83) testing negative to rAni s 1 were also negative by SPT. On the other hand, 24/46 (52.2%) and 41/46 (89.1%) sera from the sABD group tested positive for rAni s 1 and rAni s 7, respectively.

Interestingly, the statistical analysis of the allergic and sABD populations revealed significant differences (*p* < 0.001) between the percentages of sera testing positive for rAni s1 in the allergic (77.5%) versus the sABD population (52.2%), but not between the corresponding positive values for rAni s 7 in both populations (96% and 89%, respectively; Table 2b). Significant statistical differences (*p* < 0.001) were also found when comparing the percentages of sera testing positive to both recombinant allergens (double positives) in the allergic population (77.6%) versus the sABD population (52.2%) (Table 2b).

Finally, it is noteworthy that, although the OD ELISA values may be not comparable when they fall out of the linear region of the ELISA curve, the mean serum sIgE OD values in the allergic group were also significantly higher than in sABD (1.47 and 0.13 for sIgE-Ani s 1, 1.34 and 0.57 for sIgE anti-Ani s 7, respectively; *p* < 0.005; Table 2a).

### Correlation analysis between sIgE measured by ImmunoCAP and component-resolved diagnosis and clinical symptoms

A still non-solved problem in *Anisakis*-induced allergy is to know which parasite allergens, among those inducing sensitization, are responsible of the allergic symptoms showed by many patients after being parasitized. Since, as showed above, the percentage of subjects having sIgE to *Anisakis* allergens may be different in allergic and sensitized non-allergic populations, we investigated whether these differences can be related with clinical symptoms using a given cut-off.

As all analyzed sera were selected using the ImmnoCAP method, discrimination between groups was not possible at a cut-off value of ≥ 0.35 kU/L (class 1). However, when the cut-off was increased to ≥ 3.5 kU/L, a plateau in ImmunoCAP and CRD values (sIgE anti-rAni s 1 and anti-rAni s 7) was observed, suggesting that these methods could also be used to discriminate between symptomatic and asymptomatic populations (see Supplementary Fig. S2b, S2c and Table S2 online). For a sIgE to *Anisakis* cut-off of 3.5 kU/L, the obtained values for sensitivity and specificity were 84.9% and 58.8%, respectively. However, these values reached 100% when only classes 5 (50–99.9 kU/L) and 6 (≥ 100 kU/L) were considered. According to previously validated cut-off values for sIgE anti-rAni s 1 and anti-rAni s 7 (ELISA) to detect *Anisakis* sensitization, their ability to discriminate between allergic and non-allergic subjects was poor with 61.3% and 95.9% sensitivity for rAni s 1 and Ani s 7, respectively, but only 66.7% and 10.9% specificity, respectively (Table 2c).

**Figure 1:**
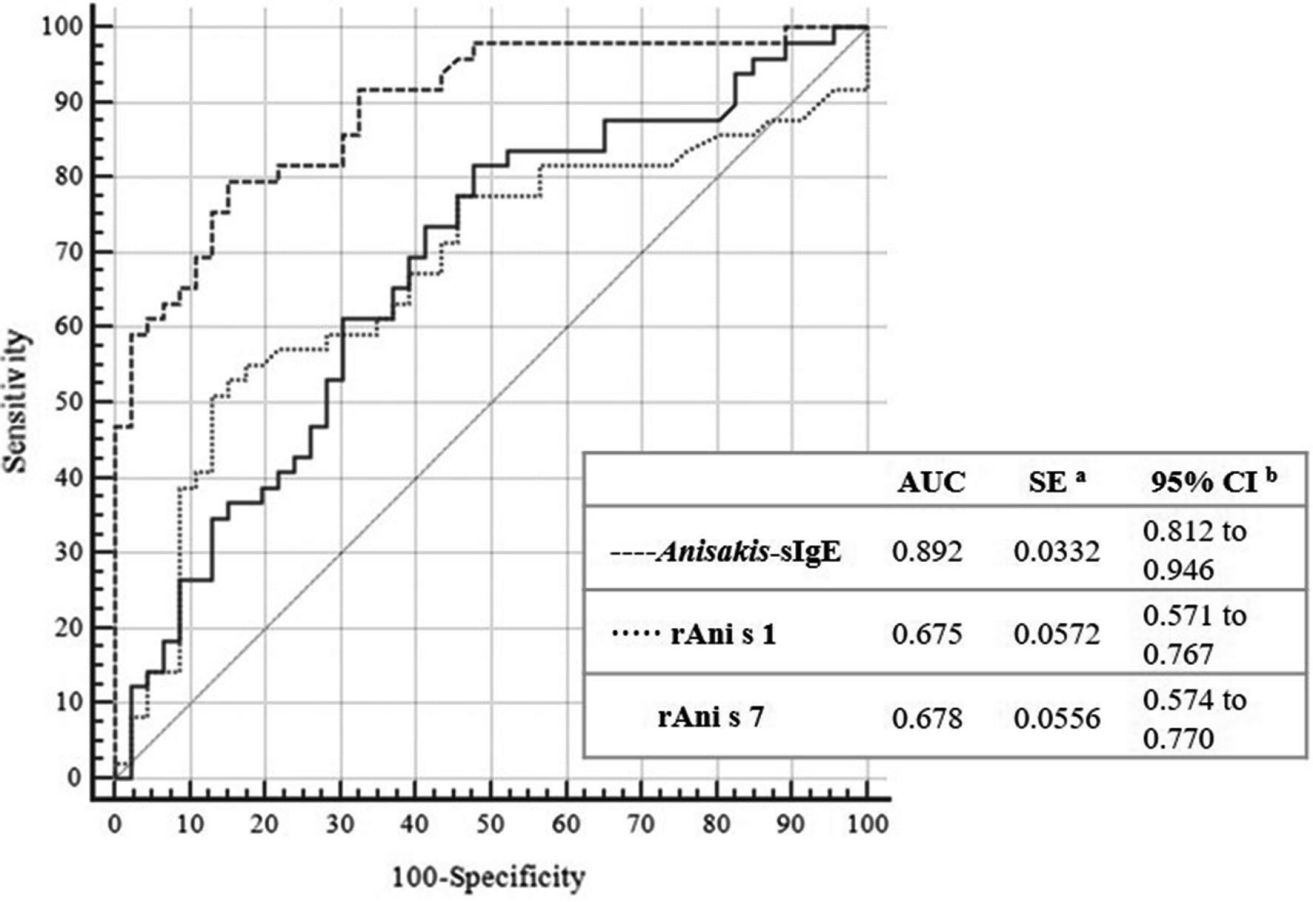
Receiver operating characteristics (ROC) curves of *Anisakis*-sIgE (InmunoCAP), rAni s 1 (ELISA) and rAni s 7 (ELISA). The sample was defined as positive group (n = 49; 51,58%), patients or blood donor with symptoms related to fish intake and as negative group (n = 46; 48,42%), sensitized blood donor without symptoms (sABD) after eating fish. Cut-off values: *Anisakis*-sIgE, 0.35 kU_A_/L; Ani s 1, 0.09; Ani s 7, 0.05. ^a^DeLong et al.^51^, ^b^binomial exact. OD, optical density.

To better evaluate the ability of Trisakis-170 recombinant allergens and ImmunoCAP to predict clinical symptoms in *Anisakis*-sensitized patients we performed a ROC analysis to calculate the better cut-off values for each allergen (Fig. 1; Table 3a). The area under the curve (AUC) for *Anisakis*-sIgE values by ImmunoCAP was 0.892, while for rAni s 1 and Ani s 7 were 0.675 and 0.678, respectively. Differences between areas were statistically significant (*p* < 0.0001; Fig. 1).

**Table 3:** Receiver operating characteristics (ROC) curves and Youden Index cut-offs of *Anisakis*-sIgE (InmunoCAP), rAni s 1 (ELISA) and rAni s 7 (ELISA). (a) Comparison of receiver operating characteristics (ROC) curves of *Anisakis*-sIgE (InmunoCAP), rAni s 1 (ELISA) and rAni s 7 (ELISA). (b) Comparison of Youden index cut-offs of *Anisakis*-sIgE (InmunoCAP), rAni s 1 (ELISA) and rAni s 7 (ELISA).

	*Anisakis*-sIgE ~ rAni s 1	*Anisakis*-sIgE ~ rAni s 7	rAni s 1 ~ rAni s 7
**(a) Comparison of ROC data**			
Difference between areas	0.217	0.214	0.00355
Standard Error^a^	0.0440	0.0403	0.0503
95% confidence interval	0.131 to 0.304	0.135 to 0.293	− 0.0951 to 0.102
z statistic	4.946	5.304	0.0705
Significance level	*p* < 0.0001	*p* < 0.0001	*p* = 0.9438

	*Anisakis-*sIgE	rAni s1	rAni s 7
**(b) Youden Index**			
Youden index J	0.6437	0.3798	0.3381
95% Confidence interval^c^	0.4689 to 0.7471	0.1996 to 0.5035	0.1603 to 0.4725
Associated criterion	> 7.9	> 1.464	> 0.589
95% Confidence interval^c^	> 1.8 to> 26.6	> 1.34 to > 1.576	> 0.053 to> 1.165
Sensitivity	79.59	51.02	81.63
Specificity	84.78	86.96	52.17

The sample was defined as positive group (n = 49; 51,58%), patients or blood donors with symptoms related to fish intake; and as negative group (n = 46; 48,42%), sensitized blood donor without symptoms after eating fish. Cut-off values: *Anisakis*-sIgE > 0.35 kU_A_/L; SPT ≥ 3 mm; rAni s 1 = 0.09 (OD); rAni s 7 = 0.05 (OD). ^a^DeLong et al.^51^, ^b^binomial exact. ^c^BC_a_ bootstrap confidence interval (1,000 iterations; random number seed: 978). OD, optical density.

When the Youden index was assessed for *Anisakis*-sIgE by ImmunoCAP the best reported cut-off point was ≥ 7.9 KUA/L showing a 79.59% sensitivity and 84.78% specificity. Positive likelihood ratio was 5.2 (IC 95:2.6–10.5) and negative likelihood ratio 0.24 (IC 95: 0.1–0.4). However, for rAni s 1 and Ani s 7 extremely high cut-offs were required (OD > 1.464 and OD > 0.589 for Ani s 1 and Ani s 7, respectively) to achieve moderate values of sensitivity and specificity (51.02% and 86.9% for rAni s 1 and 81.6% and 52.17% for rAni s 7) (Table 3b; see Supplementary Table S2 online).

**Figure 2:**
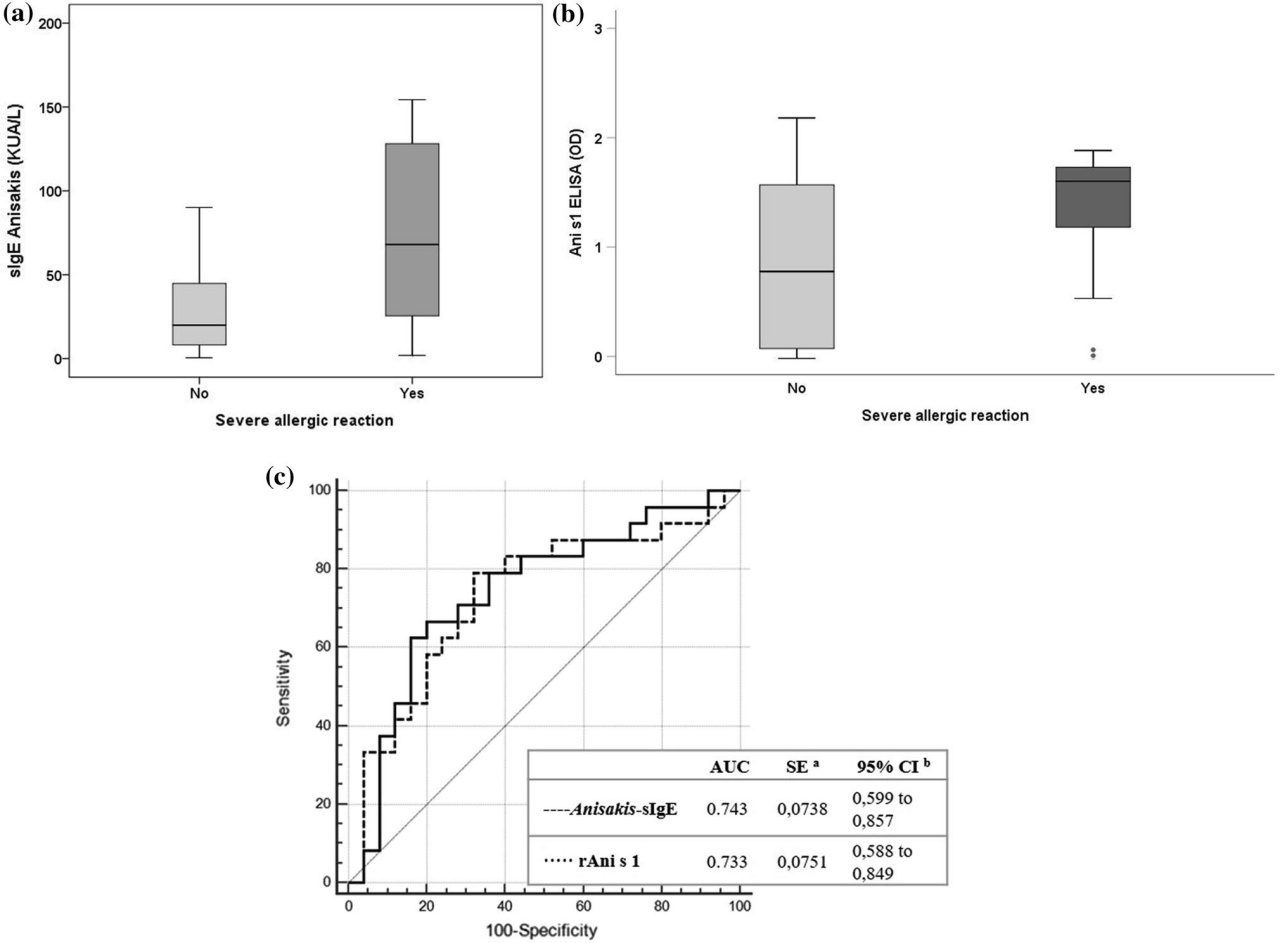
Comparation of *Anisakis*-sIgE (InmunoCAP) and rAni s 1 (ELISA) results between allergic patients who suffered a mild to moderate reaction with those who experience a severe allergic reaction. (**a**) Values of Anisakis-sIgE (mean of 0.65 and 1.61, respectively; *p* = 0.002). (**b**) Values of sIgE to rAni s 1 (mean of 15.90 and 72.60, respectively;* p* = 0.042). (**c**) Receiver operating characteristics (ROC) curves of *Anisakis*-sIgE (InmunoCAP) and rAni s 1 (ELISA) defined as positive group (n = 24; 48.98%), patients who presented a severe reaction after eating fish infected by AS and as negative group (n = 25; 51.02%), patients who presented mild to moderate reactions when eating fish infected by *A. simplex*. Cut-off values: Anisakis-sIgE > 0.35 kUA/L; Ani s 1 = 0.09. ^a^DeLong et al.^51^, ^b^binomial exact.

These results suggest that ImmunoCAP is better than CRD to predict the apparition of clinical symptoms of allergy after *Anisakis* infections. Nevertheless, the results obtained with CRD showing a better correlation of allergy symptoms with positivity to rAni s 1 versus rAni s 7 suggests that the former is more relevant in inducing allergic symptoms during *Anisakis* infections than rAni s 7. In this sense, after comparing the levels of sIgE anti-whole *Anisakis* antigens (ImmunoCAP) and anti-rAni s 1, we find significant differences in mean values between patients who have suffered a severe reaction, compared with those who experiences mild to moderate reactions (Fig. 2a, b).

### House dust mites (*Dermatophagoides pteronyssinus*) and shellfish (shrimp) sensitization in asymptomatic sensitized individuals

It is well known that *A. simplex* shares allergen epitopes with house dust mites (HDM) such as *D. pteronyssinus* (DPT), and shellfish^33^. In this study, we observed that most of sABD (43 sera) tested also for sIgE antibodies to HDM and shrimp. However, although all these presented with variable amounts of sIgE anti-DPT (range: 2.86–978 kUA/L) only 6/43 seropositive sera to shrimp (range: 0.00–3 kUA/L). In addition, a positive correlation was observed in the sABD between sIgE to DPT and shrimp with total IgE, *Anisakis*-sIgE and sIgE anti-rAni s 7 (Spearman’s Rho) (Table S3 online). Specifically, the highest R values (R ≥ 0.5) were obtained comparing total IgE versus sIgE to *Anisakis* by ImmunoCAP (R = 0.52), total IgE versus sIgE to DPT (R = 0.88), total IgE versus sIgE to shrimp (R = 0.62), sIgE to *Anisakis* by ImmunoCAP versus sIgE to rAni s 7 (R = 0.66), and sIgE to shrimp versus sIgE to DPT (R = 0.63). However, significant R values were also obtained for other combinations including Ani s 7 versus sIgE to shrimp or DTP (see Supplementary Table S3 online).

## Discussion

It is well known that anisakiasis is a parasitic infection which may course or not with associated allergic symptoms. Currently, the demonstration that an allergic patient has specific circulating IgE antibodies induced during a previous infection by *Anisakis* can be done by several in vitro techniques. However, to predict which subjects having anti-*Anisakis* IgE antibodies are at a risk of suffering allergic symptoms after a second contact with the parasite, and to dilucidate which *Anisakis* allergens are clinically relevant are questions that remain to be solved. In the last years, precision medicine applied to *A. simplex* allergy has tried to discriminate between allergy, cross reactivity and asymptomatic sensitization to this parasite through molecular diagnosis, detecting sIgE to different *A. simplex* major allergens such as Ani s 1 and Ani s 7^17,34,35^. A possible way to investigate these problems is searching for differences in the *Anisakis* antigenic profiles recognized by sensitized—allergic—versus sensitized non-allergic subjects. As the number of commercially available *Anisakis* allergens is limited, and as a proof of concept, in this study we investigated with which frequency allergic and non-allergic *Anisakis* sensitized patients recognize the Ani s 1 and Ani s 7 allergens in Cantabria.

Considering the population of BD, our results show a seroprevalence of *Anisakis*-sIgE (tested by ImmunoCAP) of 12.65% in Cantabria, which is consistent with previous studies carried out in other Spanish geographic areas^9^. No seroprevalence values were obtained for rAni s 1 and Ani s 7 since only ImmunoCAP-positive sera were tested using the Trisakis-170 test. However, sABD tested also positive by Trisakis-170 (89.13%), we estimated that a seroprevalence of 12–15% is expected in Cantabria. No significant differences between allergic patients and sABD were found in frequency of fish intake, which can be explained because Cantabria is a region with a high rate of fish consumption. On the other hand, gender and age showed differences between the group of allergic and sABD, which can be due to the fact that blood donors are usually young males meanwhile Th2-driven allergic diseases are more prevalent in females^36^ (Table 1).

When the cut-off of *Anisakis*-sIgE (tested by ImmunoCAP) was analyzed based on clinical features in allergic patients and in sABD, we observed that an increase from 0.35 to 7.9 kUA/L improved its clinical significance (likelihood ratio of 5.2–0.24). Thus, a positive test result will be 5.2 times more likely in patients with *Anisakis* allergy than in asymptomatic population, while a negative test is 4.2 times (i.e. 1/0.24) more likely in patients without *Anisakis* allergy.

Regarding the population of allergic patients, only two sera out of 49 (4.1%) tested negative by rAni s 7, while 11/49 (22.4%) tested negative by rAni s 1. In our population, no serum tested positive to rAni s 1 and negative to rAni s 7. The proportions of sensitized patients against rAni s 7 and rAni s 1 in our allergic population were similar to those previously reported for patients with gastro-allergic anisakiasis or *Anisakis*-induced chronic urticaria^17^ and confirm previous reports on the immunodominant IgE response to these allergens (mainly to Ani s 7) during the infections by *A. simplex*^28,34,37^. Like for the allergic population, the percentage of sera testing positive to Ani s 7 in the sABD group was also extremely high (41/46; 89.1%), this percentage dropped to 52.2% (24/46) for rAni s 1.

The high percentage of seropositivity to rAni s 1 in the allergic population (77.5%) and a lower value in the sABD population (52.2%), compared with the responses to rAni s 7 in both populations (95.9% and 89.1%, respectively), suggests that Ani s 1 is a clinically relevant allergen, or at least, more clinically relevant than Ani s 7. Although Ani s 7 is the most immunodominant reported *Anisakis* allergen, it also induces high levels of IgG4 antibodies during *Anisakis* acute infections (e.g. gastroallergic anisakiasis), which could have a protecting role against developing some allergic symptoms (e.g., chronic urticarial) to this allergen^38^. Moreover, the repetitive structure variants composing this secreted antigen^37^ suggests it may be involved in evasion of the immune response in the definitive natural host, for example, acting as a decoy antigen. In contrast, Ani s 1^39^ is probably of higher clinical relevance because it is structurally related with the family of Kunitz-type serin protease inhibitors largely reported as clinically-relevant allergens^27,40^.

Using a sample of 43 sera from the sABD population, we observed that all of them were also sensitized to DPT (IgE positive by ImmunoCAP) while only 6/43 (13.9%) tested positive for shrimp. As several *Anisakis* allergens (e.g. Ani s 3 and Der p 10—tropomyosin—or Ani s 2 and Der p 11—paramyosin) were reported to induce cross-reactive with mites, insects or shrimp^32,41^, a doubt arises about whether the positive correlations observed between IgE to DTP, *Anisakis* and shrimp (see Supplemental Table S3 online) are due to cross-reactions or, alternatively, that such patients were sensitized to several allergens. Although based in the present data, none of the hypothesis can be totally discarded, some data suggests that concomitant sensitizations are more probable in our cohort of patients. While cross-reacting antigens (e.g., tropomyosin), which are present in *Anisakis*, DTP and shrimp, may boost IgE antibody responses to each other thus provoking false positive results in ImmunoCAP (targeted with whole antigen mixtures), such cross-reactivity is improbable with Trisakis-170 analysis, mainly with Ani s 7. This hypothesis is supported by three facts: (1) the Ani s 7 allergen included in the kit is a polypeptide of 283 residues, which does not have relevant sequence identity with any know human allergen^37^; no cross-reactions with mites were not previously reported for this allergen, and (2) there is experimental evidence that Cantabria is a region having one of the highest allergen concentrations of *D. pteronyssinus* (ref https://alergiaweb.files.wordpress.com/2014/03/mapa-acarolc3b3gico-de-espac3b1a-de-l-leti.pdf).

Comparing the results obtained between determinations of sIgE by ImmunoCAP and Trisakis 170 only 5 sera were negative with the latter in the population of sABD. This raises the question of whether they are false sensitizations due to cross-reactivity that are frequently associated to the ImmunoCAP method or false negative results due to a small number of infected subjects that do not induce antibodies to Ani s 1/Ani s 7. Another possibility, that the test Trisakis 170 was less sensitive than ImmunoCAP seems less probable as this test detected patients with, as little as, 0.35 kUA/L by ImmnoCAP.

A preferential response to some other *Anisakis* allergens such as Ani s 4 (cystatin), Ani s 5 (SXP/RAL protein), Ani s 11 or Ani s 13 (haemoglobin)^41–43^, can explain that some sera test positive by ImmunoCAP and negative to Trisakis 170. Also, cross-reactions due to *Ascaris* infections was also reported as a possible cause of detecting sensitization to *Anisakis* in asymptomatic patients^30,44,45^, which can be related with IgE responses to pan-allergens as paramyosin (Ani s 2)^31,46^.

In summary, in this study we reported for the first time 12.65% prevalence values of *Anisakis* sensitization in Cantabria. We showed a different intensity and frequency of response to *Anisakis*-sIgE measured by ImmunoCAP and Ani s 1 by ELISA, respectively, between *A. simplex* allergic patients and asymptomatic sensitized population. Also, higher frequency of recognition of the rAni s 1 allergen was found in patients who has experienced a severe reaction compare which those who had suffered a mild to moderate one (Fig. 2).

In consequence, anti-*Anisakis* sIgE ImmunoCAP values ≥ 7.9 kUA/L and high OD signals to the rAni s 1 allergen could be potential biomarkers to recognize patients at risk of suffering severe allergic reaction after a re-infection by the parasite.

## Materials and methods

All methods were carried out in accordance with relevant guidelines and regulations.

### Study design, subjects and serum samples

A cross-sectional study with prospective data collection was performed at the Marques de Valdecilla University Hospital. The study was approved by the Clinical Research Ethics Committee of Cantabria (CEIC. Protocol number: 2016.074). All patients included in the study gave written informed consent for blood extraction, an anonymous survey about fish consumption habits and a subsequent consult if necessary.

The prevalence of IgE sensitization to *A. simplex* (*Anisakis*-sIgE > 0.35 KUA/L, ImmunoCAP) was investigated in a group of 403 BD randomly recruited at the Blood and Tissue Bank of Cantabria, Spain (control group) (see Supplementary Fig. S1 online). Among them, 51 subjects tested positive to *Anisakis* by ImmunoCAP, of which 47 individuals reported no previous allergic/gastrointestinal symptoms after fish consumption, chronic urticaria or dyspepsia (sABD group) (see Supplementary Fig. S1 online). One serum was lost during storage, so the remaining 46 sABD sera were used for further studies. Moreover, a follow up two years after the recruitment date was performed among this asymptomatic subgroup. The purpose was to verify if subjects belonging to the sABD group develop any clinical symptom after fish consumption during that time. In parallel, sera from 49 patients with allergy to *A. simplex*, were recruited for comparisons at the Allergy Department of Marques de Valdecilla University Hospital. On the inclusion day, a written survey (which included demographic data, fish consumption details and contact information) was completed by all subjects and a blood sample was collected. A Vacutainer SST II Advance (Becton Dickinson) tube was used to obtain and separate the serum sample. After a clot was formed, tubes were centrifuged at 2,000 g for 15 min at 25 °C. Serum was collected and stored at − 20 °C until use.

The group of allergic patients to *A. simplex* had experienced mild to severe allergic or gastrointestinal symptoms (urticaria, angioedema, dyspnea, nauseas, vomiting, abdominal pain, anaphylaxis) a few hours after eating suspicious *A. simplex* infected fish, presented with *Anisakis*-sIgE values greater than 0.35 KUA/L and sIgE to fish < 0.35 KUA/L^11,47^.

The sample size was determined previously assuming an *A. simplex* sensitization prevalence around 13%^9,48^ for a population of 588,656 (https://www.ine.es/jaxiT3/Datos.htm?t=2893), considering a 5% precision and a 95% confidence level, to detect a meaningful difference (20–25%) in the main variables evaluated between groups.

### Skin testing and determination of Specific IgE to *Anisakis simplex* by ImmunoCAP

All allergic patients and 21 out of 47 of the enrolled sABD underwent SPT to *A. simplex* using commercial extracts (Laboratorios LETI, Spain; 165 mcg/mL). SPTs were performed on the volar side of the forearm by using disposable 1-mm-tip lancets and were also conducted with histamine (Roxall; 10 mg/ml) as positive and saline solution (0.9% NaCl) as negative controls. Readings were taken at 20 min after application. A mean wheal diameter of ≥ 3 mm was considered positive. sIgE to whole *A. simplex* extract (*Anisakis*-sIgE) was determined by ImmunoCAP system (Thermo Fisher Scientific Inc., Uppsala, Sweden) according to the manufacturer’s instructions. A positive result was considered *Anisakis*-sIgE > 0.35 kUA/l. The same method was used to determinate total IgE, and sIgE to fish.

### Determination of specific IgE to rAni s 1 and rAni s 7 by ELISA

Fourty-six sABD and the 49 allergic patients (both groups with *Anisakis*-sIgE > 0.35 KUA/L) were analyzed by ELISA (Trisakis-170 ELISA. Parasitology Laboratory at Santiago de Compostela University, Spain) to detect sIgE-Ani s 1 and sIgE-rAni s 7 allergens, as previously described^28,49,50^. Cut off values (calculated absorbance) were considered OD = 0.09 for rAni s 1 and OD = 0.05 and rAni s 7^28,37^.

### Specific IgE to *Dermatophagoides pteronyssinus* and shrimp by InmunoCAP

sIgE to DPT and shrimp was determined by ImmunoCAP system (*Thermo Fisher *Scientific Inc., Uppsala, Sweden) in all allergic patients and in 43 sABD following the manufacture instructions.

### Statistical analysis

To analyze possible associations between demographic characteristics (independent variables) and clinically relevant sensitization to *A. simplex* (dependent variable) a univariate and multivariate logistic regression analyses were used. Statistical analysis of diagnosis test results was carried out by logistic regression analysis and Receiver Operating Characteristics (ROC) curve. A *p* value of 0.05 or less was considered significant. The SPSS (version 20.0) software package was used for all data analyses. The correlation between serum levels were analyzed by MedCalc Statistical Software version 19.1 (MedCalc Software bvba, Ostend, Belgium; https://www.medcalc.org; 2019).

### Ethics approval and consent to participate

A cross-sectional study with prospective data collection was performed at the Marques de Valdecilla University Hospital. The study was approved by the Clinical Research Ethics Committee of Cantabria (CEIC. Protocol Number: 2016.074). All patients included in the study gave written informed consent for blood extraction, an anonymous survey about fish consumption habits and a subsequent consult if necessary.

## Supplementary information

Supplementary information

## Data Availability

The datasets used and/or analyzed during the current study are available from the corresponding author on reasonable request.

## Abbreviations

*A. simplex*: *Anisakis simplex*
*Anisakis*-sIgE: Specific IgE-antibodies to *Anisakis simplex*
AUC: Area under the curve
BD: Blood donors
CRD: Component-resolved diagnosis
ELISA: Enzyme-linked immunosorbent assay
HDM: House dust mites
IgE: Immunoglobulin E
MC: Mast cell
sABD: Sensitized asymptomatic blood donors
sIgE: Specific immunoglobulin E
sIgE anti-rAni s 1: Specific IgE-antibodies to recombinant Ani s 1 allergen
sIgE anti-rAni s 7: Specific IgE-antibodies to recombinant Ani s 7 allergen
SPT: Skin prick test
rAni s 1: Recombinant Ani s 1 allergen
rAni s 7: Recombinant Ani s 1 allergen
ROC: Receiver operating curve
