# Author Correction: Analysis of Ani s 7 and Ani s 1 allergens as biomarkers of sensitization and allergy severity in human anisakiasis

**DOI:** 10.1038/s41598-020-75954-1

**Published:** 2020-10-28

**Authors:** Leticia de las Vecillas, Pedro Muñoz‑Cacho, Marcos López‑Hoyos, Vittoria Monttecchiani, Victoria Martínez‑Sernández, Florencio M. Ubeira, Fernando Rodríguez‑Fernández

**Affiliations:** 1 Department of Allergy, Marqués de Valdecilla University Hospital-Instituto de Investigación Marqués de Valdecilla (IDIVAL), Santander, Spain; 2 Department of Immunology, Marqués de Valdecilla University Hospital-Instituto de Investigación Marqués de Valdecilla (IDIVAL), Santander, Spain; 3 Gerencia Atención Primaria, Servicio Cántabro de Salud, Santander, Spain; 4 Department of Microbiology and Parasitology, Faculty of Pharmacy, University of Santiago de Compostela, Santiago de Compostela, Spain; 5 Instituto de Investigación en Análisis Químicos y Biológicos (IAQBUS), Universidad de Santiago de Compostela, Santiago de Compostela, Spain

Correction to: *Scientific Reports* https://doi.org/10.1038/s41598-020-67786-w, Published online 09 July 2020

In this Article, the legend of Figure 2a and 2b is incorrect:

“Comparation of *Anisakis*-sIgE (InmunoCAP) and rAni s 1 (ELISA) results between allergic patients who suffered a mild to moderate reaction with those who experience a severe allergic reaction. (**a**) Values of Anisakis-sIgE (mean of 0.65 and 1.61, respectively; *p* = 0.002). (**b**) Values of sIgE to rAni s 1 (mean of 15.90 and 72.60, respectively; *p* = 0.042).”

should read:

“Comparation of *Anisakis*-sIgE (InmunoCAP) and rAni s 1 (ELISA) results between allergic patients who suffered a mild to moderate reaction with those who experience a severe allergic reaction. (**a**) Values of Anisakis-sIgE (mean of 15.90 and 72.60, respectively; *p* = 0.002). (**b**) Values of sIgE to rAni s 1 (mean of 0.65 and 1.61, respectively; *p* = 0.042).”

Additionally, the Supplementary Information file that accompanies this Article contains an error in Supplementary Figure S1, where the number of total asymptomatic blood donors who underwent SPT is 20 out of 30 with a total of 12/20 positive results and not 12/30.

Furthermore, the legend for Supplementary Figure S1 is incomplete,

“Clinical and diagnostic test results in the Blood donors’ population. From 403 blood donors, 53 had positive levels of Anisakis-sIgE (≥ 0.35KUA/L, ImmunoCAP^®^). sIgE to rAni s 1 and rAni s 7 were analyzed by ELISA in 46 asymptomatic blood donors with *Anisakis*-sIgE greater than 0.35 KUA/L (n=47). A clinical follow-up were done in 31 of them and SPT were performed in 21. ^¥^Patients presenting allergic symptoms related to recent consumption of fish. ^§^Patients with urticaria, not related to recent consumption of fish. ^¤^Patients with gastrointestinal disorders not related to recent consumption of fish. ^¶^Patients without symptoms related to recent consumption of fish. CRD, component resolve diagnostic test. SPT, skin prick test.”

should read:

“Clinical and diagnostic test results in the Blood donors’ population. From 403 blood donors, 53 had positive values of Anisakis-sIgE (≥ 0.35KUA/L, ImmunoCAP^®^) from which 47 were asymptomatic. sIgE to rAni s 1 and rAni s 7 were analyzed by ELISA in 46 sensitized asymptomatic blood donors with *Anisakis*-sIgE greater than 0.35 KUA/L (n = 47). A clinical follow-up was done in 31 of them and SPT were performed in 21. ^¥^Patients presenting allergic symptoms related to the recent consumption of fish. ^§^Patients with urticaria, not related to the recent consumption of fish. ^¤^Patients with gastrointestinal disorders not related to the recent consumption of fish. ^¶^Patients without symptoms related to the recent consumption of fish. CRD, component resolve diagnostic test. SPT, skin prick test.”

The correct Figure S1 and its accompanying legend appear below as Figure 1.

**Figure 1:**
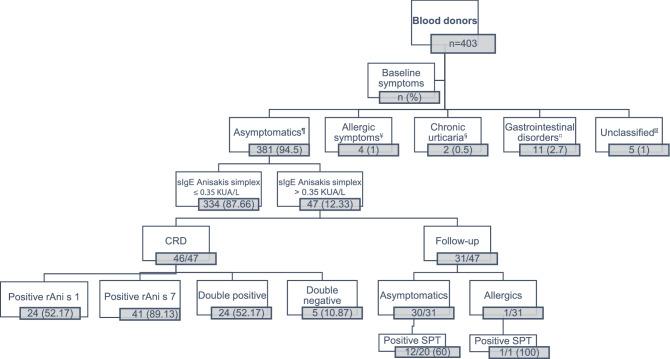
Clinical and diagnostic test results in the Blood donors’ population. From 403 blood donors, 53 had positive values of Anisakis-sIgE (≥0.35KUA/L, ImmunoCAP^®^) from which 47 were asymptomatic. sIgE to rAni s 1 and rAni s 7 were analyzed by ELISA in 46 sensitized asymptomatic blood donors with *Anisakis*-sIgE greater than 0.35 KUA/L (n=47). A clinical follow-up was done in 31 of them and SPT were performed in 21. ^¥^Patients presenting allergic symptoms related to the recent consumption of fish. ^§^Patients with urticaria, not related to the recent consumption of fish. ^¤^Patients with gastrointestinal disorders not related to the recent consumption of fish. ^¶^Patients without symptoms related to the recent consumption of fish. CRD, component resolve diagnostic test. SPT, skin prick test.

