# Supplementary material for: Analysis of Ani s 7 and Ani s 1 allergens as biomarkers of sensitization and allergy severity in human anisakiasis
Source: Sci Rep. 2020 Jul 9;10:11275. doi: 10.1038/s41598-020-67786-w (PMC7347943; doi:10.1038/s41598-020-67786-w)
Supplement: Supplementary file 1 — Supplementary information [file 41598_2020_67786_MOESM1_ESM.ppt]

## Slide 1
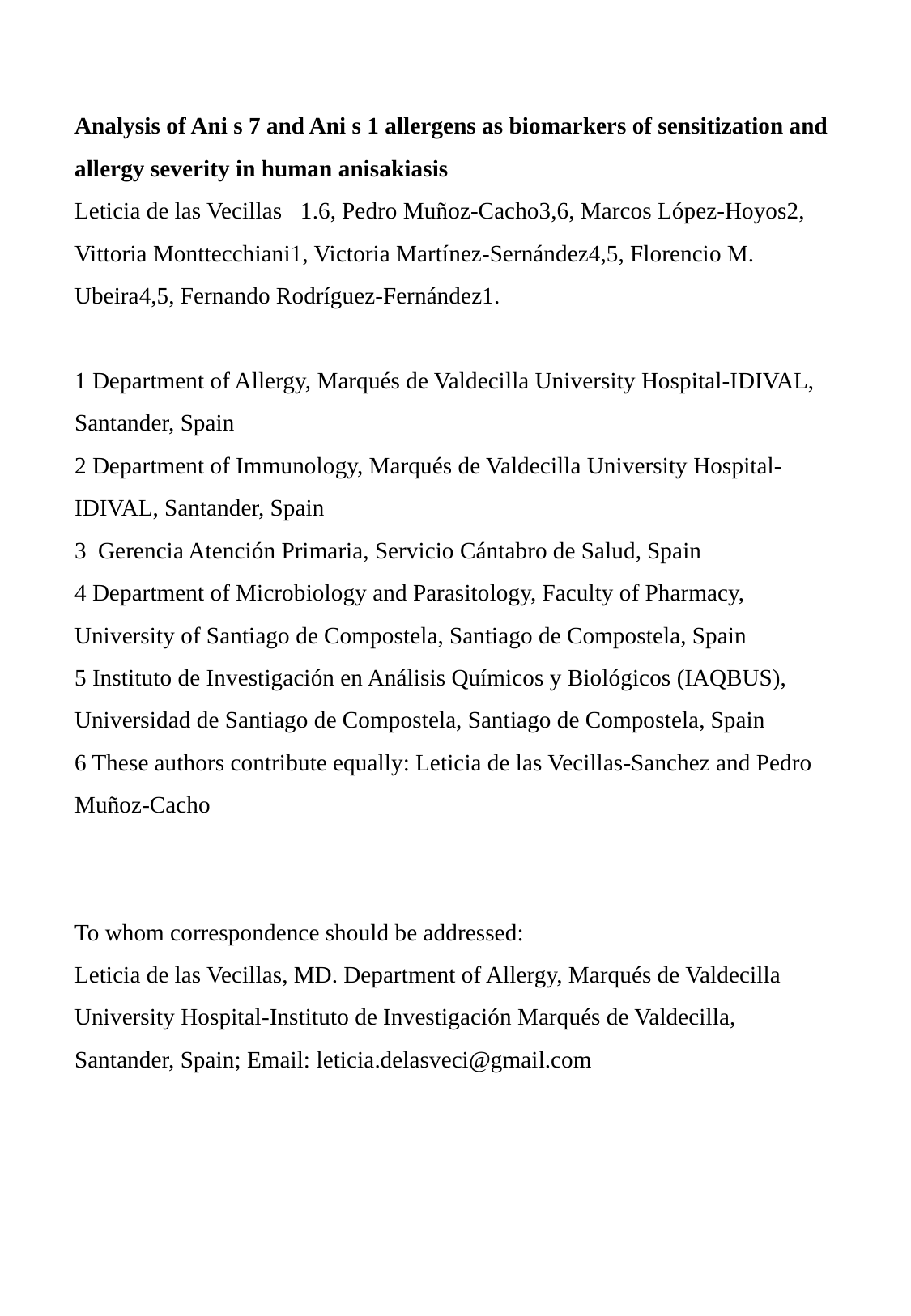

# Analysis of Ani s 7 and Ani s 1 allergens as biomarkers of sensitization and allergy severity in human anisakiasis Leticia de las Vecillas1.6, Pedro Muñoz-Cacho3,6, Marcos López-Hoyos2, Vittoria Monttecchiani1, Victoria Martínez-Sernández4,5, Florencio M. Ubeira4,5, Fernando Rodríguez-Fernández1. 1 Department of Allergy, Marqués de Valdecilla University Hospital-IDIVAL, Santander, Spain 2 Department of Immunology, Marqués de Valdecilla University Hospital- IDIVAL, Santander, Spain 3 Gerencia Atención Primaria, Servicio Cántabro de Salud, Spain 4 Department of Microbiology and Parasitology, Faculty of Pharmacy, University of Santiago de Compostela, Santiago de Compostela, Spain 5 Instituto de Investigación en Análisis Químicos y Biológicos (IAQBUS), Universidad de Santiago de Compostela, Santiago de Compostela, Spain 6 These authors contribute equally: Leticia de las Vecillas-Sanchez and Pedro Muñoz-Cacho To whom correspondence should be addressed: Leticia de las Vecillas, MD. Department of Allergy, Marqués de Valdecilla University Hospital-Instituto de Investigación Marqués de Valdecilla, Santander, Spain; Email: leticia.delasveci@gmail.com

## Slide 2
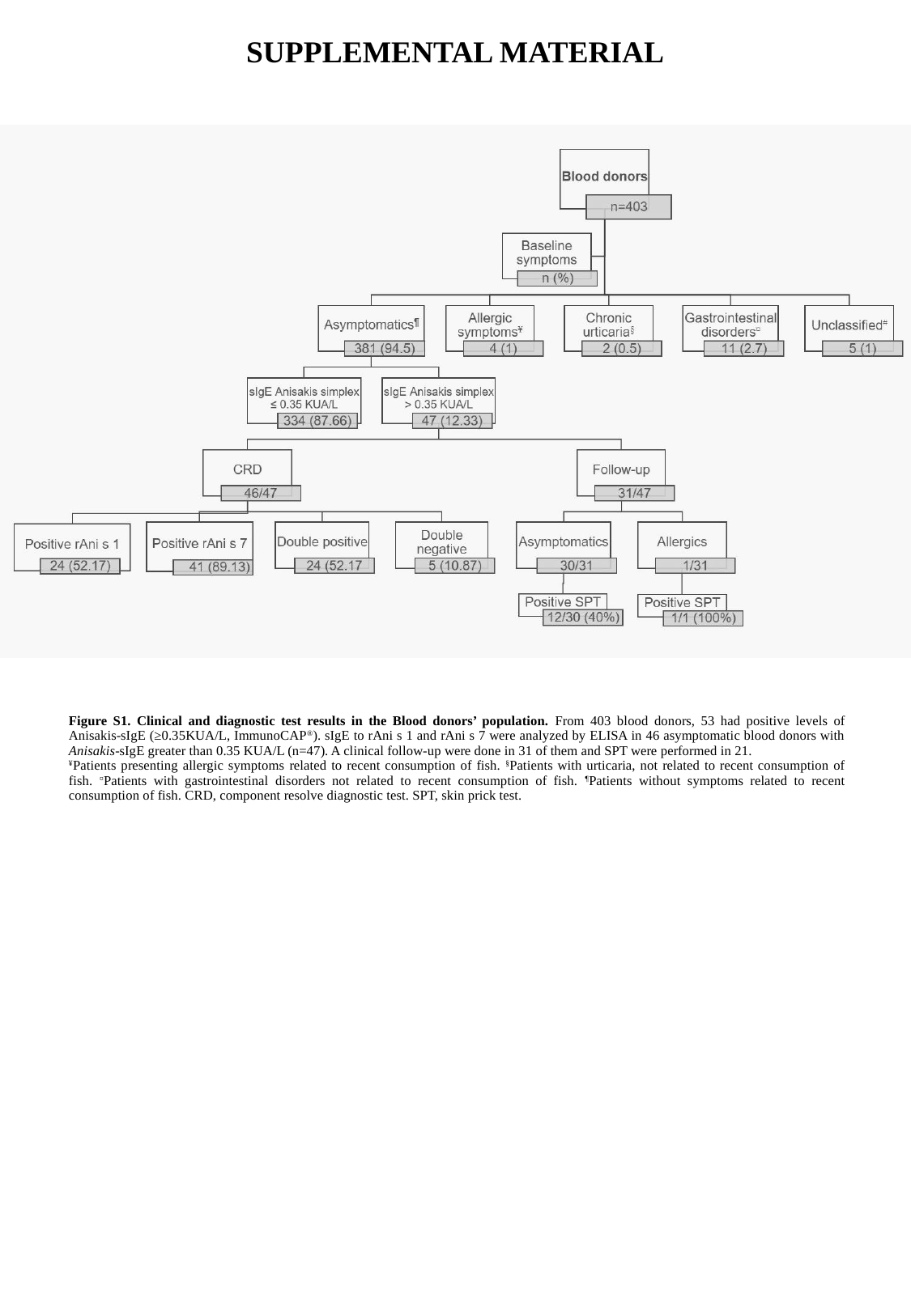

SUPPLEMENTAL MATERIAL
Figure S1. Clinical and diagnostic test results in the Blood donors’ population. From 403 blood donors, 53 had positive levels of Anisakis-sIgE (≥0.35KUA/L, ImmunoCAP®). sIgE to rAni s 1 and rAni s 7 were analyzed by ELISA in 46 asymptomatic blood donors with Anisakis-sIgE greater than 0.35 KUA/L (n=47). A clinical follow-up were done in 31 of them and SPT were performed in 21.
¥Patients presenting allergic symptoms related to recent consumption of fish. §Patients with urticaria, not related to recent consumption of fish. ¤Patients with gastrointestinal disorders not related to recent consumption of fish. ¶Patients without symptoms related to recent consumption of fish. CRD, component resolve diagnostic test. SPT, skin prick test.

## Slide 3
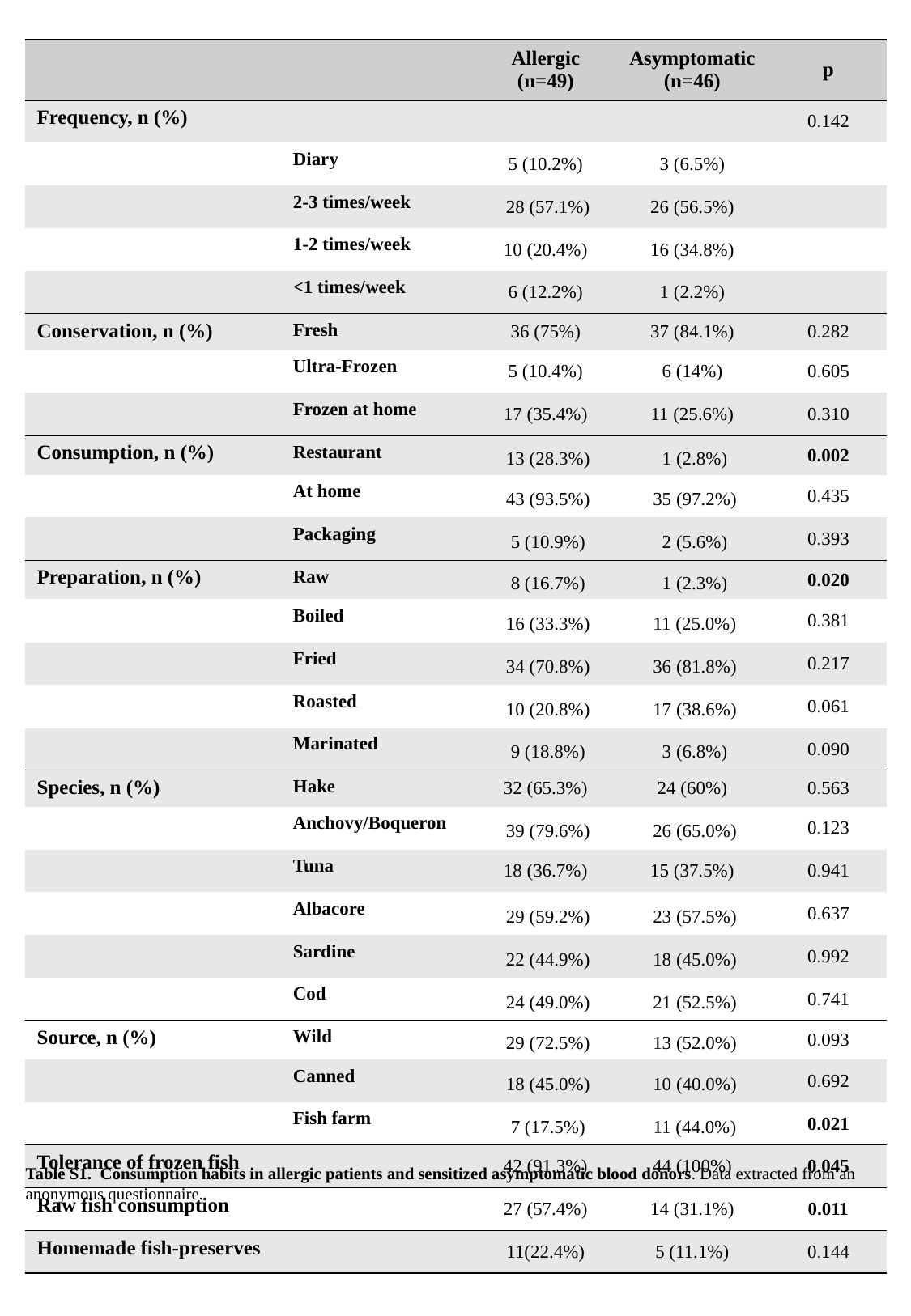

| | | Allergic (n=49) | Asymptomatic (n=46) | p |
| --- | --- | --- | --- | --- |
| Frequency, n (%) | | | | 0.142 |
| | Diary | 5 (10.2%) | 3 (6.5%) | |
| | 2-3 times/week | 28 (57.1%) | 26 (56.5%) | |
| | 1-2 times/week | 10 (20.4%) | 16 (34.8%) | |
| | <1 times/week | 6 (12.2%) | 1 (2.2%) | |
| Conservation, n (%) | Fresh | 36 (75%) | 37 (84.1%) | 0.282 |
| | Ultra-Frozen | 5 (10.4%) | 6 (14%) | 0.605 |
| | Frozen at home | 17 (35.4%) | 11 (25.6%) | 0.310 |
| Consumption, n (%) | Restaurant | 13 (28.3%) | 1 (2.8%) | 0.002 |
| | At home | 43 (93.5%) | 35 (97.2%) | 0.435 |
| | Packaging | 5 (10.9%) | 2 (5.6%) | 0.393 |
| Preparation, n (%) | Raw | 8 (16.7%) | 1 (2.3%) | 0.020 |
| | Boiled | 16 (33.3%) | 11 (25.0%) | 0.381 |
| | Fried | 34 (70.8%) | 36 (81.8%) | 0.217 |
| | Roasted | 10 (20.8%) | 17 (38.6%) | 0.061 |
| | Marinated | 9 (18.8%) | 3 (6.8%) | 0.090 |
| Species, n (%) | Hake | 32 (65.3%) | 24 (60%) | 0.563 |
| | Anchovy/Boqueron | 39 (79.6%) | 26 (65.0%) | 0.123 |
| | Tuna | 18 (36.7%) | 15 (37.5%) | 0.941 |
| | Albacore | 29 (59.2%) | 23 (57.5%) | 0.637 |
| | Sardine | 22 (44.9%) | 18 (45.0%) | 0.992 |
| | Cod | 24 (49.0%) | 21 (52.5%) | 0.741 |
| Source, n (%) | Wild | 29 (72.5%) | 13 (52.0%) | 0.093 |
| | Canned | 18 (45.0%) | 10 (40.0%) | 0.692 |
| | Fish farm | 7 (17.5%) | 11 (44.0%) | 0.021 |
| Tolerance of frozen fish | | 42 (91.3%) | 44 (100%) | 0.045 |
| Raw fish consumption | | 27 (57.4%) | 14 (31.1%) | 0.011 |
| Homemade fish-preserves | | 11(22.4%) | 5 (11.1%) | 0.144 |
Table S1. Consumption habits in allergic patients and sensitized asymptomatic blood donors. Data extracted from an anonymous questionnaire.

## Slide 4
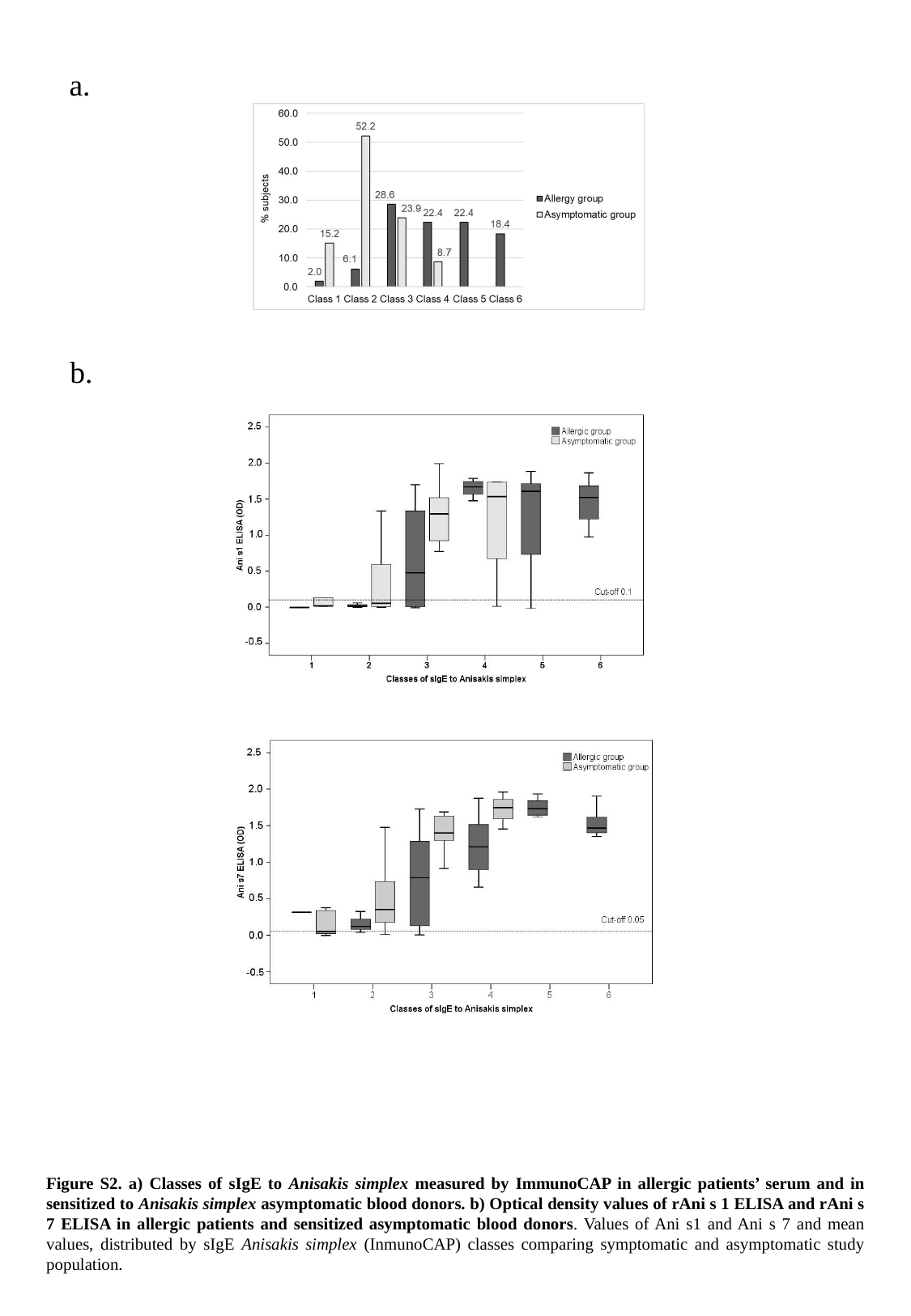

a.
b.
Figure S2. a) Classes of sIgE to Anisakis simplex measured by ImmunoCAP in allergic patients’ serum and in sensitized to Anisakis simplex asymptomatic blood donors. b) Optical density values of rAni s 1 ELISA and rAni s 7 ELISA in allergic patients and sensitized asymptomatic blood donors. Values of Ani s1 and Ani s 7 and mean values, distributed by sIgE Anisakis simplex (InmunoCAP) classes comparing symptomatic and asymptomatic study population.

## Slide 5
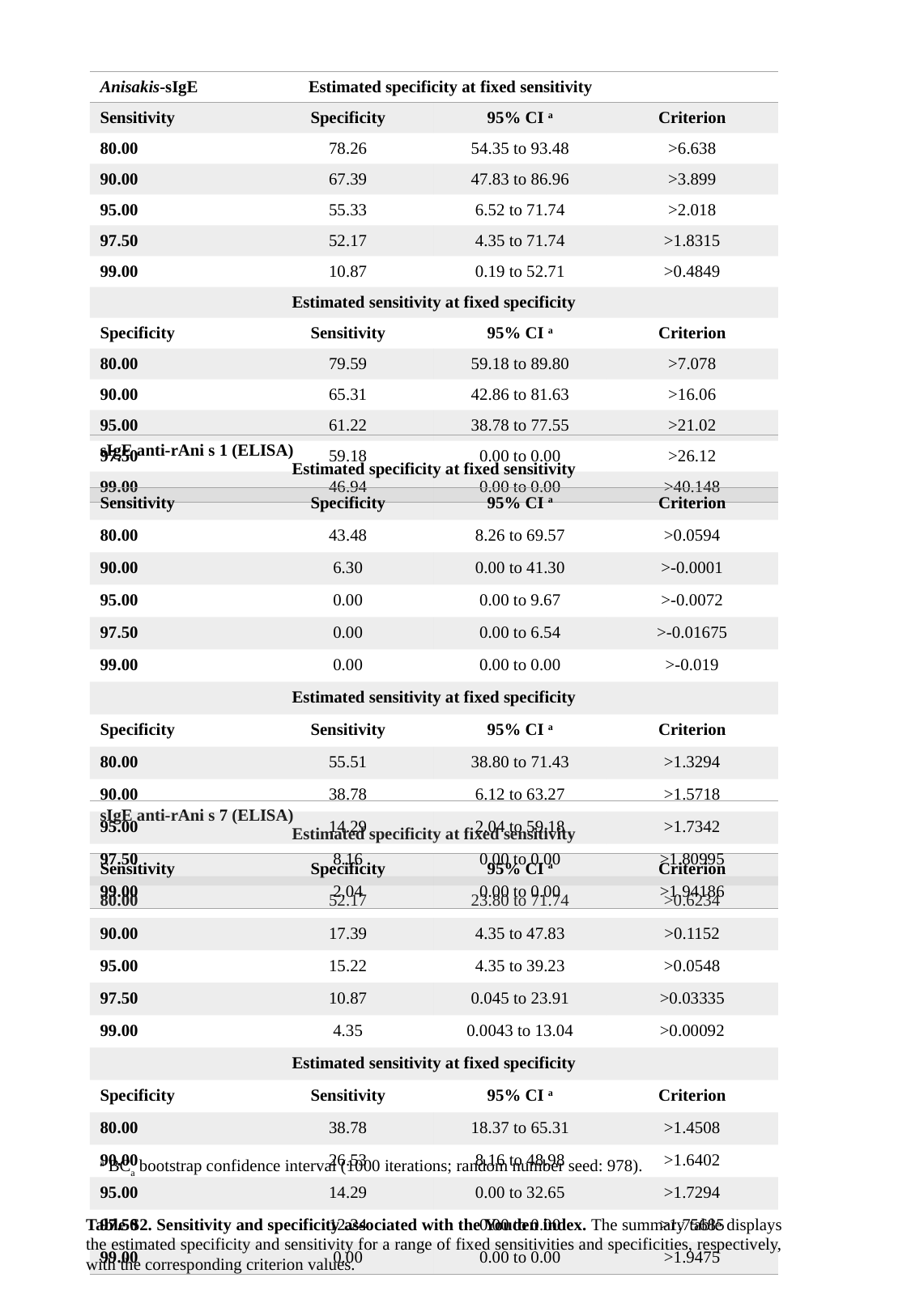

| Anisakis-sIgE Estimated specificity at fixed sensitivity | | | |
| --- | --- | --- | --- |
| Sensitivity | Specificity | 95% CI a | Criterion |
| 80.00 | 78.26 | 54.35 to 93.48 | >6.638 |
| 90.00 | 67.39 | 47.83 to 86.96 | >3.899 |
| 95.00 | 55.33 | 6.52 to 71.74 | >2.018 |
| 97.50 | 52.17 | 4.35 to 71.74 | >1.8315 |
| 99.00 | 10.87 | 0.19 to 52.71 | >0.4849 |
| Estimated sensitivity at fixed specificity | | | |
| Specificity | Sensitivity | 95% CI a | Criterion |
| 80.00 | 79.59 | 59.18 to 89.80 | >7.078 |
| 90.00 | 65.31 | 42.86 to 81.63 | >16.06 |
| 95.00 | 61.22 | 38.78 to 77.55 | >21.02 |
| 97.50 | 59.18 | 0.00 to 0.00 | >26.12 |
| 99.00 | 46.94 | 0.00 to 0.00 | >40.148 |
| sIgE anti-rAni s 1 (ELISA) Estimated specificity at fixed sensitivity | | | |
| --- | --- | --- | --- |
| Sensitivity | Specificity | 95% CI a | Criterion |
| 80.00 | 43.48 | 8.26 to 69.57 | >0.0594 |
| 90.00 | 6.30 | 0.00 to 41.30 | >-0.0001 |
| 95.00 | 0.00 | 0.00 to 9.67 | >-0.0072 |
| 97.50 | 0.00 | 0.00 to 6.54 | >-0.01675 |
| 99.00 | 0.00 | 0.00 to 0.00 | >-0.019 |
| Estimated sensitivity at fixed specificity | | | |
| Specificity | Sensitivity | 95% CI a | Criterion |
| 80.00 | 55.51 | 38.80 to 71.43 | >1.3294 |
| 90.00 | 38.78 | 6.12 to 63.27 | >1.5718 |
| 95.00 | 14.29 | 2.04 to 59.18 | >1.7342 |
| 97.50 | 8.16 | 0.00 to 0.00 | >1.80995 |
| 99.00 | 2.04 | 0.00 to 0.00 | >1.94186 |
| sIgE anti-rAni s 7 (ELISA) Estimated specificity at fixed sensitivity | | | |
| --- | --- | --- | --- |
| Sensitivity | Specificity | 95% CI a | Criterion |
| 80.00 | 52.17 | 23.80 to 71.74 | >0.6234 |
| 90.00 | 17.39 | 4.35 to 47.83 | >0.1152 |
| 95.00 | 15.22 | 4.35 to 39.23 | >0.0548 |
| 97.50 | 10.87 | 0.045 to 23.91 | >0.03335 |
| 99.00 | 4.35 | 0.0043 to 13.04 | >0.00092 |
| Estimated sensitivity at fixed specificity | | | |
| Specificity | Sensitivity | 95% CI a | Criterion |
| 80.00 | 38.78 | 18.37 to 65.31 | >1.4508 |
| 90.00 | 26.53 | 8.16 to 48.98 | >1.6402 |
| 95.00 | 14.29 | 0.00 to 32.65 | >1.7294 |
| 97.50 | 12.24 | 0.00 to 0.00 | >1.75685 |
| 99.00 | 0.00 | 0.00 to 0.00 | >1.9475 |
a BCa bootstrap confidence interval (1000 iterations; random number seed: 978).
Table S2. Sensitivity and specificity associated with the Youden index. The summary table displays the estimated specificity and sensitivity for a range of fixed sensitivities and specificities, respectively, with the corresponding criterion values.

## Slide 6
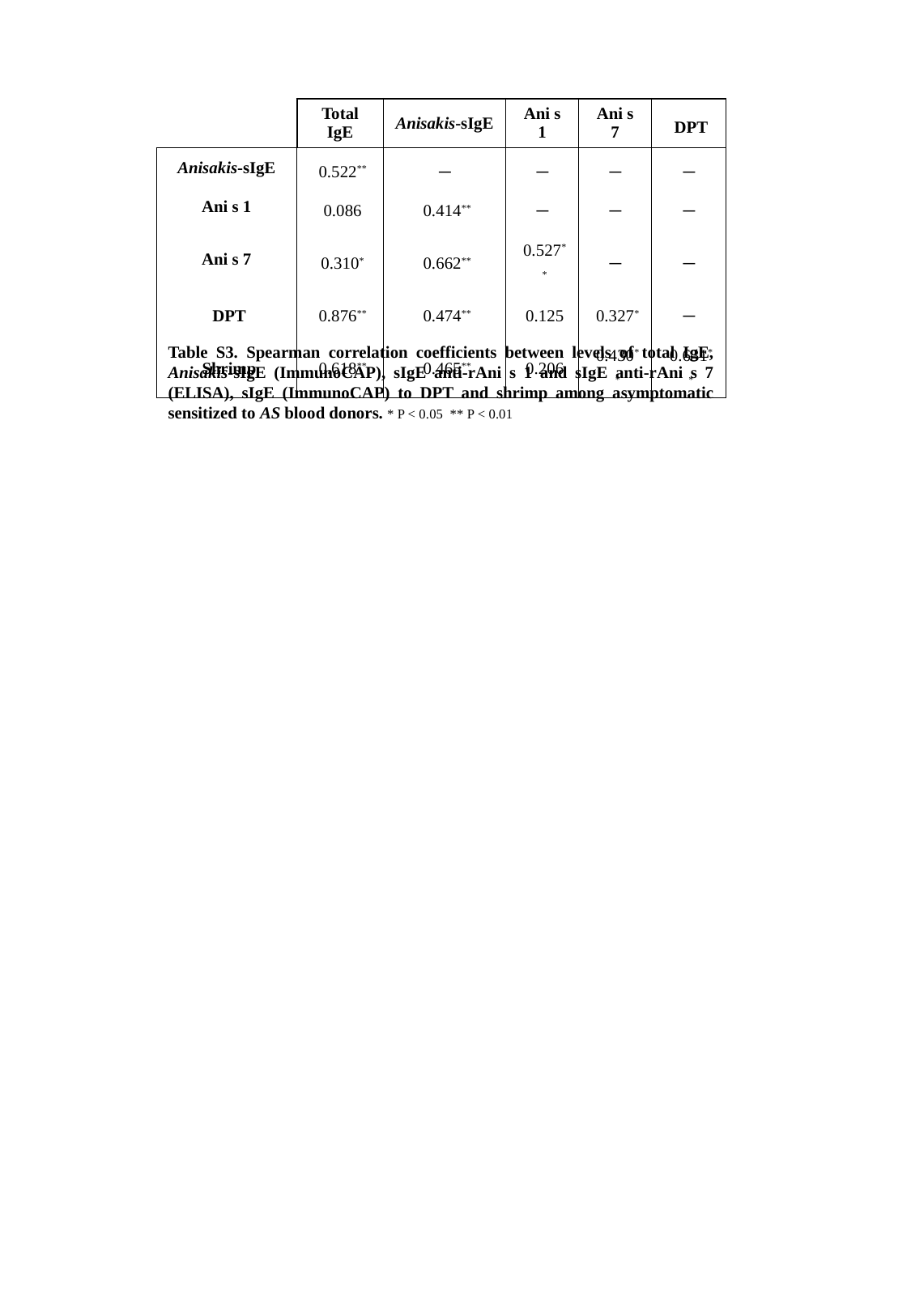

| | Total IgE | Anisakis-sIgE | Ani s 1 | Ani s 7 | DPT |
| --- | --- | --- | --- | --- | --- |
| Anisakis-sIgE | 0.522\*\* | ─ | ─ | ─ | ─ |
| Ani s 1 | 0.086 | 0.414\*\* | ─ | ─ | ─ |
| Ani s 7 | 0.310\* | 0.662\*\* | 0.527\*\* | ─ | ─ |
| DPT | 0.876\*\* | 0.474\*\* | 0.125 | 0.327\* | ─ |
| Shrimp | 0.618\*\* | 0.465\*\* | 0.206 | 0.430\*\* | 0.631\*\* |
Table S3. Spearman correlation coefficients between levels of total IgE, Anisakis-sIgE (ImmunoCAP), sIgE anti-rAni s 1 and sIgE anti-rAni s 7 (ELISA), sIgE (ImmunoCAP) to DPT and shrimp among asymptomatic sensitized to AS blood donors. * P < 0.05 ** P < 0.01
